# Chondroitin sulfate restores muscle mass via gut–muscle axis remodeling through sugar–bile acid metabolism reprogramming

**DOI:** 10.1002/imt2.70118

**Published:** 2026-03-12

**Authors:** Ruiyun Wu, Tao Wen, Nan Shang, Penghao Xie, Zhenyu Wang, Hang Li, Shaobo Li, Dequan Zhang

**Affiliations:** ^1^ Institute of Food Science and Technology, Chinese Academy of Agricultural Sciences, Integrated Laboratory of Processing Technology for Chinese Meat and Dish Products Ministry of Agriculture and Rural Affairs Beijing China; ^2^ Jiangsu Provincial Key Lab for Organic Solid Waste Utilization, Jiangsu Collaborative Innovation Center for Solid Organic Wastes, Educational Ministry Engineering Center of Resource‐saving fertilizers Nanjing Agricultural University Nanjing China; ^3^ College of Engineering China Agricultural University Beijing China; ^4^ Key Laboratory of Biomaterials of Guangdong Higher Education Institutes, Department of Biomedical Engineering Jinan University Guangzhou China

**Keywords:** chondroitin sulfate, glucocorticoid‐induced myopathy, gut–muscle axis, Lactobacillus johnsonii, multi‐omics integration, NAD⁺ metabolism

## Abstract

Glucocorticoid‐induced myopathy is characterized by progressive muscle atrophy and impaired regeneration, yet effective microbiota‐oriented interventions for preserving muscle homeostasis remain largely unexplored. Here, we demonstrate that dietary chondroitin sulfate (DCS) restores muscle mass and function through a microbiota‐dependent gut–muscle metabolic axis. DCS failed to confer protection in germ‐free or antibiotic‐treated mice, establishing gut microbiota as a prerequisite for its efficacy. Microbiota transplantation and mono‐colonization experiments identified *Lactobacillus johnsonii* Z‐RW as a functionally relevant mediator capable of recapitulating muscle protection under controlled microbial conditions. Integrated metagenomic, metabolomic, and proteomic analyses revealed coordinated reprogramming of intestinal sugar utilization and bile acid metabolism following DCS administration. Notably, DCS promoted bile acid deconjugation and enrichment of secondary bile acids, coinciding with restoration of muscle regenerative and energetic programs, including upregulation of NMRK2, PAX7, and SIRT1. Metabolite supplementation further implicated bile acids as candidate mediators linking microbial metabolism to muscle phenotypes. To quantitatively integrate these shifts, we introduce the sugar‐bile acid ratio as a systems‐level descriptor of microbiota‐driven metabolic remodeling. Our findings delineate a microbiota‐dependent metabolic framework through which a functional polysaccharide reshapes intestinal biochemistry to influence distal muscle physiology. This work highlights bile acid‐associated signaling as a central relay within the gut‐muscle axis and provides a conceptual foundation for microbiota‐targeted strategies to mitigate muscle wasting.

## Highlights

- Chondroitin sulfate (DCS) reverses muscle atrophy by remodeling gut–axis.
- Enriched *L*. *johnsonii* Z‐RW bile salt hydrolase reprograms gut sugar‐bile acid metabolism.
- Z‐RW activates PAX7/NMRK2‐mediated muscle regeneration.

## Graphical abstract

The graphical abstract illustrates the molecular mechanism by which chondroitin sulfate (DCS) alleviates glucocorticoid‐induced myopathy through the gut–muscle axis. DCS selectively enriches *L. johnsonii* Z‐RW, enhancing *bshA*‐encoded BSH activity to promote bile acid deconjugation and reduce the sugar–bile acid ratio, thereby re‐establishing intestinal metabolic homeostasis. The resulting secondary bile acids cross the intestinal barrier and reach muscle tissue, where they activate NMRK2‐mediated NAD⁺ biosynthesis and PAX7 signaling, upregulating myogenic proteins such as KRT18, MYHC, and RGN. These coordinated molecular events restore energy metabolism and muscle function.

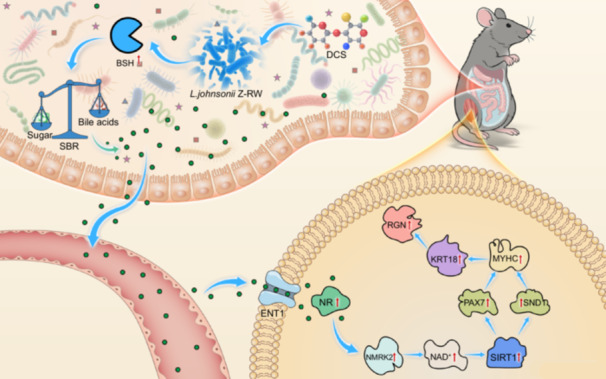

## INTRODUCTION

Glucocorticoids (e.g., dexamethasone) remain clinically irreplaceable for controlling systemic lupus erythematosus, organ transplant rejection, and malignant tumors due to their potent immunosuppressive and anti‐inflammatory effects [1]. However, long‐term use of dexamethasone induces severe muscle atrophy via the gut–muscle axis, a bidirectional link increasingly implicated in human metabolic and immune diseases. This axis is disrupted by glucocorticoid‐driven dysbiosis, elevated ubiquitin‐proteasome activity, mitochondrial dysfunction, and impaired satellite cell function, manifesting as proximal muscle wasting, myofiber type shift, and reduced protein synthesis [2, 3]. Current therapeutic strategies mainly include dose reduction or discontinuation, combined with rehabilitation and nutritional support. However, these approaches present significant challenges: long‐term users often struggle to maintain full muscle function, while patients dependent on glucocorticoids for disease control (e.g., in systemic lupus erythematosus, cancer, or transplant rejection) cannot discontinue treatment. Given that glucocorticoid‐induced muscle damage often involves dysfunction of multiple pathways, such as imbalanced energy metabolism, inhibition of protein synthesis, and elevated oxidative stress [4], there is an urgent need for the development of targeted interventions that can be implemented over a long term without compromising the efficacy of the steroid. In contrast to the mode of action of conventional drugs that act directly on muscle fiber structures, nutritional intervention modulates metabolic pathway activity, enhances cellular homeostatic regulation, and has low toxicity. These properties have made it a potentially safe strategy for maintaining muscle function and mitigating side effects.

Recent studies have shown that nutritional intervention targeting metabolic pathways is gradually becoming an important research direction for alleviating the muscle side effects associated with glucocorticoids due to its ability to regulate metabolic homeostasis, low toxicity, and long‐term sustainability [5]. In particular, some non‐digestible carbohydrates (e.g., oligosaccharides, polysaccharides, and glycosaminoglycans) that can be selectively utilized by intestinal flora can be metabolized by specific microbes to produce biologically active small‐molecule metabolites, which can affect the energy metabolism, protein homeostasis, and muscle function of the host [6, 7]. Chondroitin sulfate (CS) is a structurally well‐defined and widely available natural glycosaminoglycan composed of repeating units of N‐acetylgalactosamine and glucuronic acid, and it demonstrates significant metabolic and immunomodulatory effects [8]. Animal experiments showed that CS intervention effectively reduced serum AChR antibody levels and improved muscle motility, with changes in intestinal flora structure and intestinal mucosal barrier function, in an experimental autoimmune myasthenia gravis model. In addition, human intestinal strains such as *Bacteroides salyersiae* have been shown to efficiently degrade CS to produce unsaturated chondroitin sulfate oligosaccharides and short‐chain fatty acids (SCFAs) [9]. These in turn contribute to the regulation of local intestinal immune homeostasis and may modulate the metabolic status of distal tissues through a cross‐barrier mechanism [7, 10].

In this context, chondroitin sulfate (DCS) featuring a distinctive 2‐position sulfation pattern was obtained from animal bone‐derived resources and characterized by its high solubility and metabolic stability. Our previous work demonstrated that DCS supplementation effectively alleviated both glucocorticoid‐ and denervation‐induced muscle atrophy, highlighting its potential as a bioactive polysaccharide for muscle protection. However, the molecular mechanism through which DCS mitigates muscle atrophy by modulating the gut microbiota and its metabolites remains largely unexplored.

Although the gut–muscle axis has emerged as a critical framework linking intestinal metabolism to skeletal muscle homeostasis, the specific cascade from microbial metabolites to host signaling pathways and muscle phenotypes has yet to be elucidated. In this study, we systematically investigated the mechanism of DCS action in a dexamethasone‐induced myopathy mouse model by integrating histological assessment, 16S rRNA sequencing, and targeted metabolomics. Our study aims to elucidate (1) whether DCS improves muscle function by reshaping intestinal microecology; (2) how DCS‐mediated metabolic alterations regulate signaling pathways in muscle tissue; and (3) whether these effects along the gut–muscle axis are dose‐dependent, strain‐specific, and contingent on specific signaling pathways.

## RESULTS

### Chondroitin sulfate restores muscle mass and function in dexamethasone‐treated mice

**Figure 1:**
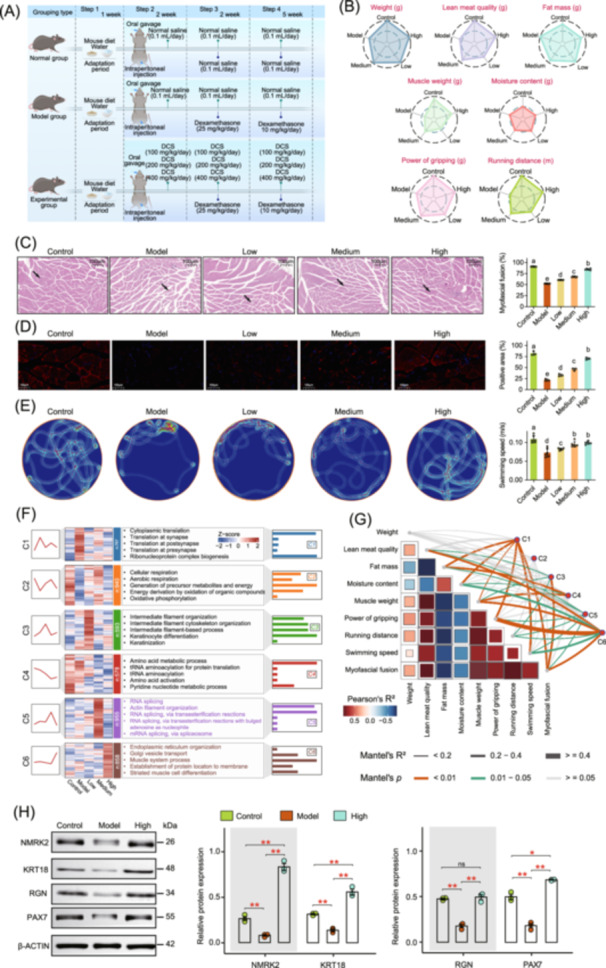
DCS enhances muscle regeneration and functional performance through remodeling of protein expression networks in myopathic mice. (A) Schematic diagram of the experimental procedure in mice. (B) Radar chart showing the improvement of muscle function in mice after DCS treatment. Each treatment group includes five replicates. (C) H&E staining of gastrocnemius muscle in mice under different treatments, the bar graph illustrates the improvement in gastrocnemius muscle function in mice following different DCS treatments. (data are presented as mean ± SD). Arrows indicate regions of muscle fiber atrophy, characterized by reduced fiber diameter. Different lowercase letters indicated significant differences among respective groups based on ANOVA test for multiple comparisons by Tukey's HSD corrections (*p* < 0.05, *n* = 6 biologically independent replicates). (D) Representative immunofluorescence staining of gastrocnemius muscle in mice across different treatment groups (Control, Model, Low, Medium, and High). Myosin heavy chain (MHC, red) and nuclei (blue) are shown. The bar graph on the right quantifies the proportion of MHC‐positive fibers. (data are presented as mean ± SD). Different lowercase letters indicated significant differences among respective groups based on ANOVA test for multiple comparisons by Tukey's HSD corrections (*p* < 0.05, *n* = 3 biologically independent replicates). (E) Presentative infrared swimming trajectories of mice under different treatments (Control, Model, Low, Medium, and High). Trajectories illustrate both swimming distance and velocity, where warmer colors (red) indicate longer residence time at a given location, corresponding to slower movement. The bar graph on the right shows quantitative analysis of swimming speed (m/s). (data are presented as mean ± SD). Different lowercase letters indicated significant differences among respective groups based on ANOVA test for multiple comparisons by Tukey's HSD corrections (*p* < 0.05, *n* = 6 biologically independent replicates). (F) Heatmap displaying scaled expression of representative proteins enriched in mouse muscle clusters (C1–C6; left), alongside GO term enrichment analysis for specific regions, highlighting associated molecular functions and biological processes (right). (G) Mantel test results between muscle function indicators and protein expression modules. Mantel's r and *p* values are represented by the color and width of connecting lines, as detailed in the figure legend. Pearson correlation coefficients (two‐sided) were computed for muscle function. Significance levels: ns, *p* ≥ 0.05; *p < 0.05; **p < 0.01. (H) Representative Western blot bands and corresponding densitometric quantification of NMRK2, KRT18, RGN, and PAX7 protein expression in the indicated groups, with β‐ACTIN used as the loading control. Bar graph illustrates protein expression levels across different groups (data presented as mean ± SD, *n* = 3 biologically independent replicates). Group differences were assessed by one‐way ANOVA with Tukey's HSD post hoc test (*p* < 0.05). Significance levels: ns, *p* ≥ 0.05; *p < 0.05; **p < 0.01.

To evaluate the potential of chondroitin sulfate (DCS) in alleviating the symptoms of myopathy, myopathy was induced in mice using dexamethasone, followed by DCS intervention (Figure 1A). Its effects were evaluated by muscle function assessment and histological analysis. The results showed that the DCS intervention significantly improved several muscle function‐related indicators in a favorable dose‐dependent manner. The mice in the high‐dose group significantly outperformed those in the model group in muscle weight, forelimb grip strength, running distance, swimming speed, and lean mass (Figure 1B and Table S1). To assess whether DCS exerts a microbiota‐independent direct effect on skeletal muscle, we examined its systemic distribution and direct cellular activity. Following oral administration, DCS exhibited low exposure in the circulation and peripheral tissues, with no evidence of pronounced tissue accumulation (Table S2). At concentrations corresponding to the maximal in vivo muscle exposure, DCS treatment did not significantly affect cell viability or the expression of MyoD and MyHC in differentiated C2C12 myotubes (Figure S1A).

To further confirm the synergistic repair effect of DCS on muscle tissue structure, mouse gastrocnemius muscle was stained with HE for analysis, and the results showed that DCS effectively protected and rebuilt the microstructure of muscle fibers (Figure 1C). Subsequently, immunofluorescence staining was used to quantitatively analyze a hallmark indicator of the degree of muscle atrophy. The proportion of MHC positivity was 82.37% in the normal control group, and sharply decreased to 21.95% in the model group. After DCS intervention, the proportion of MHC positivity increased in a dose‐dependent manner, and it significantly increased to 70.29% in the high‐dose group, which was close to the normal level (Figure 1D). The results of the pool‐stay test showed that the heat map of the dexamethasone model group demonstrated a limited range of motion and decreased exercise tolerance, and exercise pattern significantly improved with increasing doses of DCS intervention (Figure 1E).

Proteomic profiling of gastrocnemius muscle tissues was conducted across all experimental groups. The results showed significant differences in protein expression profiles between the different treatment groups (Figure S2). Through cluster analysis, the differential proteins were categorized into six major functional modules (Figure S3), of which the C6 module was significantly enriched in biological pathways closely related to muscle functions as revealed in gene ontology (GO) enrichment analysis, including myofiber repair, muscle cell differentiation, and energy metabolism regulation (Figure 1F).

The Mantel test showed that the C6 module was significantly and positively correlated with several muscle indices, such as muscle mass and exercise capacity (Figure 1G). Based on the C6 module, potential key regulatory proteins related to muscle function were screened out, including Nmrk2, Pax7, and Krt18, which have important roles in muscle regeneration, cell differentiation, and structural maintenance (Table S3). Western Blot validation showed that the expression levels of Nmrk2, Pax7, and Krt18 were significantly upregulated in the gastrocnemius muscle of mice in the DCS intervention group (Figure 1H, Figure S4).

### *L. johnsonii* Z‐RW was enriched and exhibits a high capacity for DCS degradation and utilization

**Figure 2:**
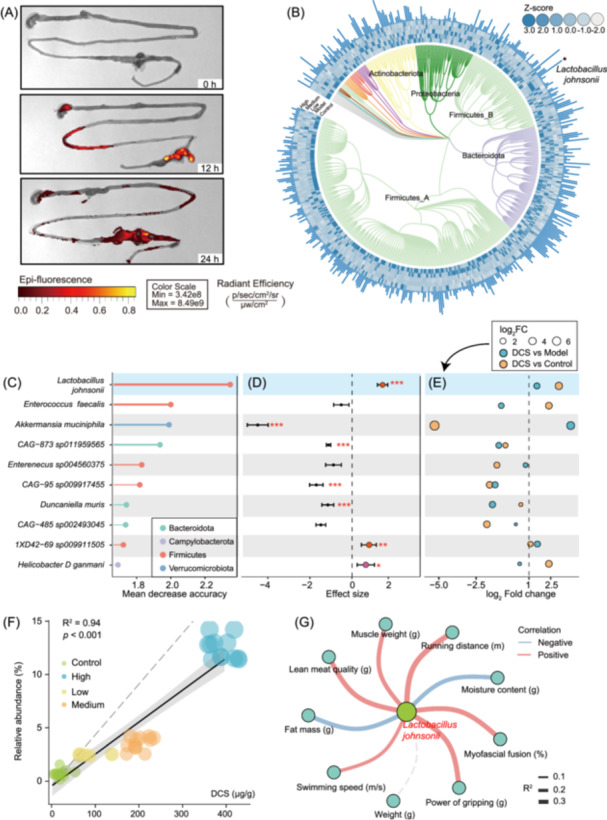
DCS reshapes gut microbiota and links microbial changes to muscle functional outcomes. (A) Fluorescence imaging of mouse intestine at 0 h, 12 h, and 24 h after oral administration of fluorescently labeled DCS. Pseudo‐color scale indicates radiant efficiency, with warmer colors denoting stronger fluorescence signals, demonstrating the time‐dependent accumulation and intestinal distribution of the compound. (B) Phylogenetic tree of species with associated abundance profiles under different treatments. The circular tree depicts the taxonomic relationships among species. The outer heatmap shows the relative abundance variation of each species across the different treatments, with color gradients representing differences in abundance. From inside to outside, the heatmap layers represent Control, Model, and Low, Medium, and High treatments. The bar plot on the periphery displays the average relative abundance of each species. (C) Top 10 microbial features identified by random forest analysis based on mean decrease in accuracy. (D) Meta‐analysis forest plot comparing the abundance of 10 microbial features between the DCS and control groups. The DCS treatment group represents the combined data from the low, medium, and high concentration treatments. Significance levels: *p < 0.05; **p < 0.01; ***p < 0.001. (E) Differential abundance of 10 microbial features in DCS‐treated mice relative to controls. The DCS treatment group represents the combined data from the low, medium, and high concentration treatments. (F) Scatter plots depicting the correlation between chondroitin sulfate concentration and the relative abundance of *L. johnsonii*. Significant correlations (*p* < 0.05) are indicated. (G) Correlation network analysis between the relative abundance of *Lactobacillus johnsonii* and murine muscle function metrics. Line thickness corresponds to the strength of the correlation; red and blue lines represent positive and negative associations, respectively.

To clarify the effector targets of DCS, in vivo imaging was used to track its in vivo distribution. The results showed that DCS passed stably through the gastrointestinal tract after gavage and was significantly enriched in the small intestine and colon. At 0 h, the fluorescent signals were only distributed in the stomach; by 10 h, some of the signals had extended to the small intestine and cecum region, and high‐intensity fluorescent aggregation was observed in the distal intestinal tract (especially in the colon) at 24 h, making it possible to directly contact and be utilized by intestinal microorganisms (Figure 2A). Importantly, acute oral administration of DCS at a dose tenfold higher than that used in the efficacy studies did not result in detectable intestinal histopathological alterations, nor did it affect body weight or food intake in mice (Figure S1B and Table S4). Consistently, DCS showed no adverse effects on the viability of normal human colonic epithelial cells (NCM460) within the physiologically relevant concentration range (Figure S1C). The changes in the gut microbiome of each group were analyzed in depth by 16S rRNA sequencing. Diversity showed a dose‐dependent U‐shaped trend after DCS intervention, with high doses of DCS significantly restoring diversity (Figure S5). The results of non‐metric multidimensional scaling (NMDS) showed that the gut flora structure was significantly altered in both the model and DCS intervention groups (STRESS value = 0.12, ANOSIM R = 0.98, *p* = 0.001), with the DCS intervention group showing greater similarity to the control group (Figure S6). A total of 502 microorganisms were identified in the phylogenetic tree, with the top 5 microorganisms in relative abundance including *Dubosiella* sp. 000403415, *Faecalibaculum rodentium*, *Paramuribaculum intestinale*, and *Lactobacillus johnsonii*, and *Alloprevotella* sp. 002933955 (Figure 2B). To screen for the core strains driving phenotypic myopathy remission, a random forest classification model was used, which had 99% model accuracy based on the top 10 characterized microorganisms (Figure S7). These microorganisms included *Lactobacillus johnsonii*, *Enterococcus faecalis*, *Akkermansia muciniphila_D*, *CAG‐873* sp. 011959565, and *Enterenecus* sp. 004560375 (Figure 2C). DCS showed positive regulatory effects on *L. johnsonii*, *1XD42‐69* sp. 009911505, and *Helicobacter_D ganmani*, whereas negative effects were observed for the other characteristic bacteria (Figure 2D). Fold‐change analysis showed that the abundance of *L. johnsonii* was significantly higher in the drug‐treated group than in both the model group (log_2_ FC = 1.69) and the normal group (log2 FC = 3.59) (Figure 2E), suggesting that it is a potential specific DCS‐responsive target strain. Regression analysis further showed that the relative abundance of *L. johnsonii* was significantly and positively correlated with DCS dose (Figure 2F, *R* = 0.94, *p* < 0.001). Cross‐domain network analysis showed that the abundance of *L. johnsonii* was significantly and positively correlated with key motor function indices such as muscle weight, running distance, swimming speed, and grip strength, and negatively correlated with fat mass and water content (*p* < 0.05; Figure 2G).

**Figure 3:**
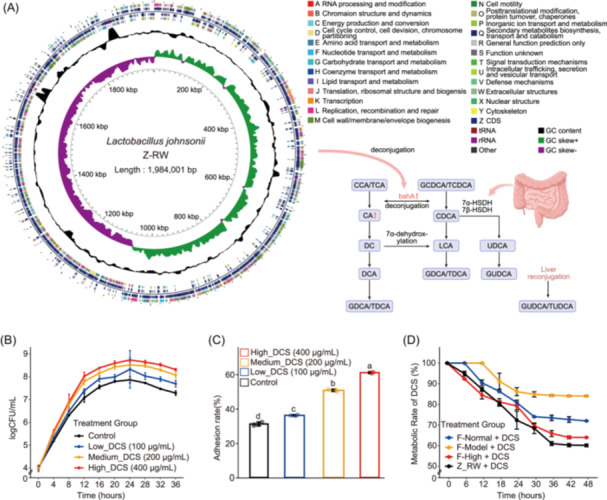
Dose‐dependent effects of DCS on growth dynamics, adhesion behavior, and metabolic responses of *L. johnsonii* Z‐RW. (A) Genomic overview of the isolated *L. johnsonii* Z‐RW strain. The circular map displays predicted coding sequences (CDSs) on the forward and reverse strands, colored by COG functional categories, with tRNA and rRNA loci indicated. Inner tracks show GC content and GC skew (positive and negative), reflecting genome‐wide nucleotide composition and replication‐associated asymmetry; genome size is labeled in base pairs. The schematic pathway diagram in the right lower panel summarizes bile‐acid biotransformation reactions potentially supported by Z‐RW (e.g., deconjugation of conjugated bile acids via bile salt hydrolase and downstream conversions involving 7α/7β‐hydroxysteroid dehydrogenases), and is included to provide a mechanistic framework linking the genomic potential of this strain to the DCS‐associated shifts in bile‐acid profiles observed in metabolomic analyses. (B) Growth kinetics of *L. johnsonii* Z‐RW cultured in media containing different concentrations of chondroitin sulfate (DCS). Data are presented as mean ± SD (*n* = 3 biologically independent replicates). (C) Effect of different DCS concentrations on the adhesion rate of *L. johnsonii* Z‐RW. Data are presented as mean ± SD. Different lowercase letters indicate significant differences among groups based on one‐way ANOVA with Tukey's HSD post hoc test (*p* < 0.05; *n* = 3 biologically independent replicates). (D) Degradation kinetics of DCS over 48 h in different fermentation systems. F denotes mouse fecal fermentation inocula derived from the indicated groups (F‐Normal, F‐Model, and F‐High), each incubated with DCS. Z‐RW + DCS represents fermentation with the isolated *L. johnsonii* Z‐RW strain supplemented with DCS. Values are presented as mean ± SD.

We isolated and purified the *L. johnsonii* strain from the fecal samples of mice in the high‐dose DCS group and designated it *L. johnsonii* Z‐RW (CGMCC 32588). The high‐quality circular genome of *L. johnsonii* Z‐RW was obtained with PacBio and Illumina hybrid sequencing, and COG functional annotations showed that *L. johnsonii* Z‐RW contained a total of 19 COG functional categories (Table S5). The typical bile salt hydrolase‐encoding gene, *bshA*, was annotated in the *L. johnsonii* Z‐RW genome and mapped to COG class I (lipid transport and metabolism). The bile salt hydrolase (BSH), the encoded product of this gene, catalyzes the hydrolysis of the amide bond of bound bile acids (e.g., taurocholic acid (TCA), and taurodeoxycholic acid (TDCA)) to produce free primary bile acids and amino acids (Figure 3A). To verify the ability of *L. johnsonii* Z‐RW and the ability of DCS to recruit it, its proliferation kinetics, DCS utilization efficiency, and intestinal adhesion potential were systematically assessed in a simulated colonic environment using DCS as the sole inducer. The results showed that, with increasing DCS concentrations, *L. johnsonii* Z‐RW exhibited a shortened lag phase and an earlier onset of exponential growth, accompanied by a significant increase in viable counts (log_10_ CFU/mL; *p* < 0.01; Figure 3B). In Caco‐2 adhesion assay, the adhesion rate rose from 30.2% in the absence of DCS to 35.8%, 50.0%, and 60.4% at 0.5, 1.0, and 2.0 mg/mL DCS, respectively (*p* < 0.01; Figure 3C). In DCS‐utilization assay, *L. johnsonii* Z‐RW exhibited a markedly faster and more extensive degradation than the high‐dose fecal microbiota and other fermentation systems, demonstrating that it is the key target bacterium responsible for driving efficient DCS metabolism in the high‐dose group (Figure 3D).

### Bile acids were enriched and promoted skeletal muscle repair, whereas small molecule sugars drove its deterioration

**Figure 4:**
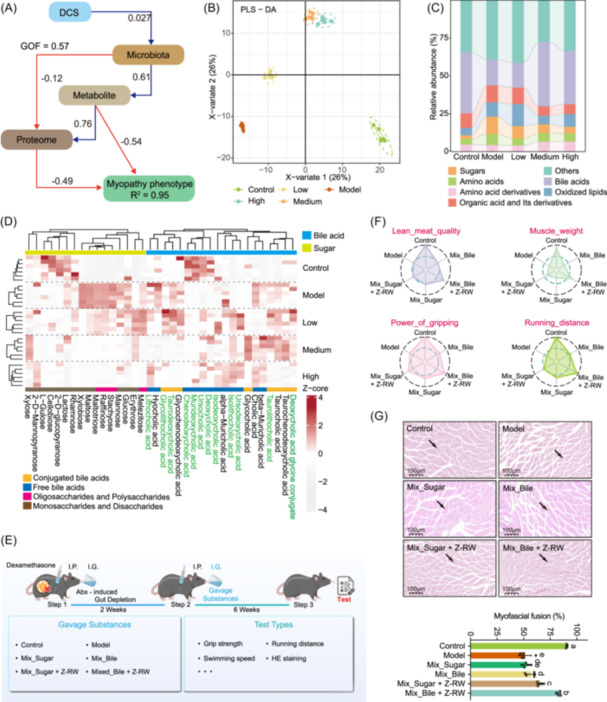
Integrated multi‐omics analysis identifies key gut metabolites mediating muscle phenotypes and validates their functional effects in mice. (A) PLS‐PM illustrating the interrelationships between the gut microbiome, gut metabolome, muscle proteome, and muscle phenotype as mediated by DCS. (B) Partial least squares‐discriminant analysis (PLS‐DA) score plot demonstrating the separation across different treatment groups. (C) Stacked bar chart showing the relative abundance changes of metabolites across groups. (D) Heatmap displaying scaled relative abundance of sugars and bile acids in the mouse intestine. Metabolite names shown in green indicate the subset of differentially altered metabolites that were highlighted for downstream analyses, whereas names in black denote the remaining detected metabolites included for clustering and visualization. (E) Schematic diagram of the mouse oral gavage experiment for key metabolites. Mice were assigned to the indicated groups throughout the entire experiment. All mice underwent a 2‐week modeling phase consisting of dexamethasone challenge by intraperitoneal injection (I.P.) with concomitant antibiotic (Abx)‐induced gut microbiota depletion by intragastric administration (I.G.), followed by a 6‐week intervention phase with daily I.G. gavage. Sterile normal saline was used as the vehicle throughout. Control mice received sterile normal saline only; Model mice received dexamethasone during the modeling phase and sterile normal saline throughout. After model establishment, gavage treatments were divided into two categories: sugar‐related treatments, including Mix_sugars (xylobiose, mannose, erythrose, and xylose) and Mix_sugars + Z‐RW (the sugar mixture combined with Z‐RW); and bile acid‐related treatments, including Mix_bile (taurodeoxycholic acid, taurocholic acid, glycochenodeoxycholic acid, and hyocholic acid) and Mix_bile + Z‐RW (the bile acid mixture combined with Z‐RW) (*n* = 5 biologically independent replicates). (F) Radar charts showing the improvement of muscle function in mice after oral administration of key metabolites. Each treatment group includes five replicates. (G) Representative H&E staining of gastrocnemius muscle in mice from different groups (Control, Model, Mix_Sugar, Mix_Sugar + Z‐RW, Mix_Bile, and Mix_Bile + Z‐RW). Arrows indicate regions of muscle fiber atrophy, characterized by reduced fiber diameter. The bar graph below shows the quantification of myofascial fusion (%) in mice after oral administration of key metabolites (data are presented as mean ± SD). Different lowercase letters indicated significant differences among respective groups based on the ANOVA test for multiple comparisons by Tukey's HSD corrections (*p* < 0.05, *n* = 5 biologically independent replicates). PLS‐PM, partial least squares path modeling.

To further elucidate the pathways through which *L. johnsonii* Z‐RW regulates skeletal muscle repair, we performed untargeted metabolomic analysis, and the structural equation model was constructed based on metabolome, microbiome, and proteome and showed good overall goodness of fit (GOF = 0.57), with DCS directly influencing the microbial community (path coefficient = 0.027), and changes in the community indirectly modulating the muscle phenotype through the metabolites (path coefficient = 0.61) and the proteome (path coefficient = 0.49), ultimately explaining a high degree of the muscle phenotype with *R*² = 0.95 (Figure 4A).

Further analysis of the untargeted metabolomics data was performed, and in the principal least squares‐discriminant analysis (PLS‐DA) plot, the treatments were significantly separated, with gradient distribution of the low, medium, and high‐dose groups where the higher the dose, the closer it was to the control group, indicating a significant dose‐dependence in the effect of the intervention (Figure 4B). Stacked histograms showed that the content of bile acids was significantly increased and the content of sugars was significantly decreased with DCS treatment (low, medium, and high) compared to the model group (Figure 4C). All compounds in the bile acids and sugar categories were further extracted for hierarchical clustering. The relative abundance of conjugated bile acids, including TDCA, glycochenodeoxycholic acid (GCDCA), and TCA, was significantly elevated in the model group compared to the control. However, treatment with a high dose of DCS reversed this trend and increased the levels of free bile acids, such as ursodeoxycholic acid (UDCA), ursocholic acid (UCA). In contrast, sugar metabolites like melezitose, erythrose, and stachyose were markedly reduced in the high‐dose treatment group (Figure 4D).

To mechanistically separate direct metabolite effects from *L. johnsonii* Z‐RW dependent metabolic remodeling, we established a structured supplementation system in sterile mice. Mixed saccharides (xylobiose, mannose, erythrose, and xylose), as well as representative bile acids (TDCA, GCDCA, TCA, and HCA), were orally administered with or without *L. johnsonii* Z‐RW (Figure 4E). Compared with the model group, mice receiving the combined bile acid inoculation and *L. johnsonii* Z‐RW intervention exhibited notable improvements in several motor phenotypes, including swimming speed and muscle mass, as well as in tissue structure, such as reduced myofascial fusion (Figure 4F and Table S6). H&E staining of the gastrocnemius further revealed that the therapeutic effect in the group treated with combined bile acids and *L. johnsonii* Z‐RW closely resembled that of the normal group, showing marked restoration of muscle fiber architecture (Figure 4G).

### The conversion of primary bile acids into secondary bile acids promoted by *L. johnsonii* Z‐RW and reduces the levels of small‐molecule sugars

Network analysis showed that the relative abundance of *L. johnsonii* was negatively correlated with bound bile acids, such as TDCA, GCDCA, and was positively correlated with free bile acids, such as isodeoxycholic acid (ISODCA), ursodeoxycholic acid (UDCA), and glycolithocholic acid (GLCA), as well as was significantly and negatively correlated with a variety of small‐molecule sugars (lactose, rhamnose, and xylose) (Figure S8). As the metabolite‐supplementation experiment revealed that the deconjugation and conversion of primary bile acids, as well as the reduction of small‐molecule sugars, were strongly associated with metabolic remodeling mediated by *L. johnsonii* Z‐RW, we further conducted direct validation of the substrate utilization and biochemical conversion capacities of *L. johnsonii* Z‐RW.

**Figure 5:**
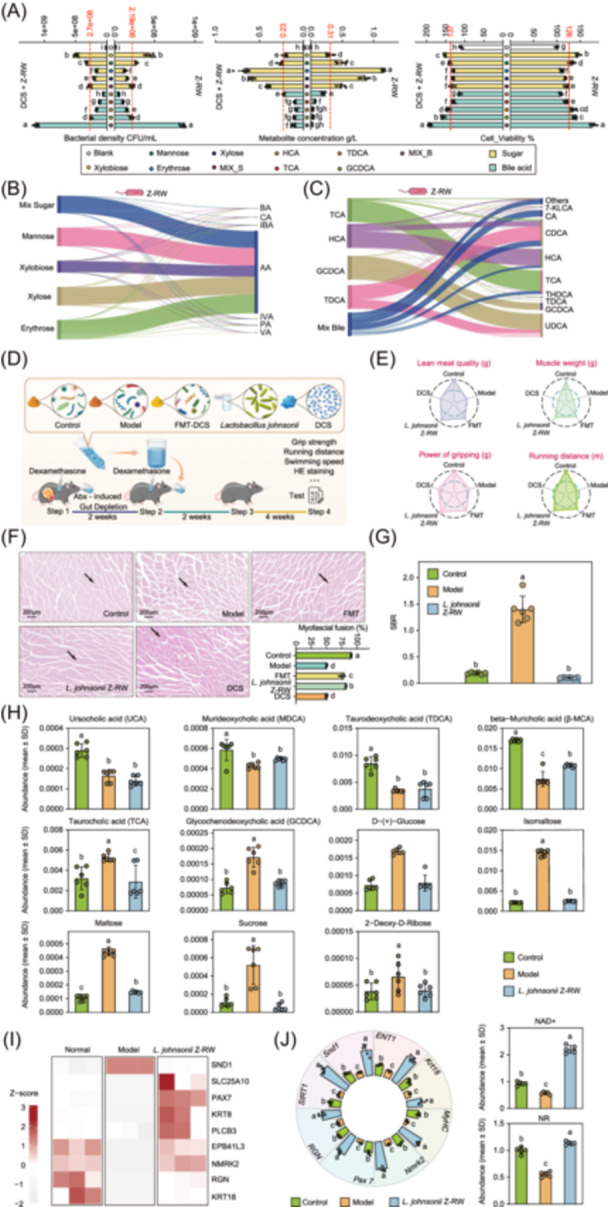
*L. johnsonii* Z‐RW metabolizes gut sugars and bile acids and improves muscle function. (A) From left to right: growth of *L. johnsonii* Z‐RW during the logarithmic phase in media containing different sugars or bile acids; residual substrate concentrations in the culture medium after metabolism by *L. johnsonii* Z‐RW; and C2C12 myotube viability after treatment with conditioned media derived from *L. johnsonii* Z‐RW cultured with different metabolites. Data are presented as mean ± SD (n = 5 biologically independent replicates). (B) Sankey diagram showing the metabolic products generated by *L. johnsonii* Z‐RW from four individual sugars and their mixture. Abbreviations: AA, acetic acid; PA, propionic acid; BA, butyric acid; IBA, isobutyric acid; VA, valeric acid; IVA, isovaleric acid; CA, caproic acid. (C) Sankey diagram showing the metabolic products generated by *L. johnsonii* Z‐RW from four primary bile acids and their mixture. (D) Schematic illustration of the experimental design for oral administration of *L. johnsonii* Z‐RW and fecal microbiota transplantation (FMT) in mice. (E) Radar chart showing muscle function improvement in mice after oral administration of *L. johnsonii* Z‐RW or FMT (*n* = 5 biologically independent samples per group). (F) Improvement in gastrocnemius muscle function in mice treated with *L. johnsonii* Z‐RW or FMT. Representative H&E‐stained sections of gastrocnemius muscle from the indicated groups are shown on the left (scale bar, 200 μm), and quantification of myofiber fusion (%) is shown on the right. Mice received *L. johnsonii* Z‐RW, FMT, or DCS as indicated. Arrows indicate areas of muscle fiber atrophy characterized by reduced fiber diameter. Data are presented as mean ± SD. Different lowercase letters indicate significant differences among groups according to one‐way ANOVA followed by Tukey's HSD post hoc test (*p* < 0.05; *n* = 5 biologically independent samples). (G) Sugar‐to‐bile acid ratio (SBR) across different treatment groups. Data are presented as mean ± SD (*n* = 5 biologically independent replicates). (H) Relative levels of major gut metabolites after *L. johnsonii* Z‐RW treatment. Data are presented as mean ± SD. Different lowercase letters indicate significant differences among groups according to one‐way ANOVA followed by Tukey's HSD test (*p* < 0.05; *n* = 6). (I) Expression levels of key proteins in response to *L. johnsonii* Z‐RW treatment. (J) Expression levels of core genes after *L. johnsonii* Z‐RW treatment. Data are presented as mean ± SD. Different lowercase letters indicate significant differences among groups according to one‐way ANOVA followed by Tukey's HSD test (*p* < 0.05; *n* = 5 biologically independent replicates).

The results showed that supplementation with DCS markedly promoted the proliferation of *L. johnsonii* Z‐RW, with a 0.5–1.0 log10 CFU/mL increase at 12 h compared with the control (Figure 5A). This was accompanied by accelerated substrate utilization, reflected by a reduction in residual sugar concentration of 0.10–0.25 g/L. In parallel, conditioned media derived from DCS‐stimulated *L. johnsonii* Z‐RW cultures promoted C2C12 myotube viability to 137–143% of the control (Figure 5A). In the five bile acid culture systems, DCS similarly increased *L. johnsonii* Z‐RW abundance by 0.3–0.6 log10 CFU/mL, reduced residual bile acids to 0.08–0.15 g/L, and further improved myocyte viability to 150–158% (Figure 5A). These results indicate that DCS preferentially enhances the growth and metabolic activity of *L. johnsonii* Z‐RW under defined substrate conditions, supporting its role as a major metabolic responder to DCS exposure.

Metabolic and transformational capabilities were further validated using absolute quantification and targeted metabolomics. The results of the incubation showed that all monosaccharides and their mixture were significantly depleted after fermentation, accompanied by an increase in the content of SCFAs such as acetic acid (Figure 5B). In terms of bile acid transformation, conjugated primary bile acids such as TCA and GCDCA were decreased, while secondary bile acids including TDCA, HCA, 7‐ketolithocholic acid (7‐KLCA), and ursodeoxycholic acid (UDCA) were increased (Figure 5C). In summary, *L. johnsonii* Z‐RW exhibits potent sugar‐fermentation and bile‐acid‐deconjugation capabilities, with pronounced efficiency in converting bound primary bile acids into secondary products. These metabolic traits position *L. johnsonii* Z‐RW as a central effector through which DCS orchestrates the remodeling of the sugar–bile acid–muscle metabolic axis, thereby linking gut microbial activity to host muscle regeneration.

### Strain transplantation combined with multi‐omics recapitulates the DCS‐triggered gut–muscle axis signaling pathway

To investigate the functional contribution of *L. johnsonii* Z‐RW in alleviating glucocorticoid‐induced myopathy, a germ‐free recipient–based strain transplantation and fecal microbiota transplantation (FMT) model was established, including an additional group of germ‐free mice receiving DCS gavage alone without microbial colonization (Figure 5D). Notably, germ‐free mice receiving DCS alone showed no appreciable improvement in muscle functional performance or morphology, as assessed by grip strength, running distance, muscle weight, swimming speed, and histological features of the gastrocnemius muscle (Figure 5E,F). In contrast, transplantation of fecal microbiota derived from DCS‐treated donor mice resulted in a partial restoration of muscle functional parameters, whereas transplantation with *L. johnsonii* Z‐RW produced the most pronounced recovery across all evaluated indices (Figure 5E and Table S7). Consistently, histological analysis revealed that *L. johnsonii* Z‐RW transplantation markedly improved gastrocnemius muscle architecture, with more regularly aligned muscle fibers and tissue morphology approaching that of control mice (Figure 5F). Collectively, these results indicate that DCS alone is insufficient to confer muscle protection in the absence of gut microbiota. In contrast, microbiota altered by DCS, particularly colonization with *L. johnsonii* Z‐RW, are associated with restoration of muscle structure and function. Accordingly, subsequent multi‐omics analyses were focused on experimental conditions in which microbial colonization or transplantation was accompanied by recovery of muscle phenotypes.

Subsequently, proteomic and metabolomic analyses were performed to assess whether *L. johnsonii* Z‐RW improved muscle function under microbiota‐reconstituted conditions by modulating intestinal metabolites and restoring the expression of key proteins. Accordingly, we proposed the “sugar‐bile acid ratio” (SBR) as a quantitative indicator of metabolic status, that is, the ratio of the abundance of small molecule sugars to free secondary bile acids (SBR = total sugars/total free secondary bile acids), and the results showed that this ratio was 0.18 in normal mice, increased to 1.1 in the model group, and decreased to 0.11 in the strain transplantation group (Figure 5G), suggesting that a decrease in the SBR associated with phenotypic improvement in microbiota‐reconstituted mice (Figure 5G). Differential metabolite analysis showed that the relative abundance of conjugated primary bile acids such as GCDCA and TCA, and sugar metabolites (D‐(+)‐glucose, maltose, isomaltose, and sucrose) were significantly decreased in the strain‐treated group compared to the model group (Figure 5H). Notably, 2‐deoxy‐d‐ribose, which was elevated in the model group, was also reduced after strain inoculation to a level comparable to the control. In contrast, the relative abundance of secondary bile acids, such as TDCA, murideoxycholic acid (MDCA), and beta‐muricholic acid (β‐MCA), was significantly increased in the strain‐inoculated group than in the model group, showing a partial restoration trend toward the control levels (Figure 5H). Meanwhile, ursodeoxycholic acid (UCA) remained reduced in both the model and strain‐treated groups relative to the control, indicating that not all secondary bile acid species were fully recovered by strain inoculation (Figure 5H). This was consistent with the results of previous analyses (Figure 4D), suggesting an active role in remodeling bile acid metabolism following effective microbial transplantation.

Proteomic analysis further revealed that inoculation of *L. johnsonii* Z‐RW significantly upregulated key proteins regulating muscle stem cell activation and NAD⁺ regeneration, such as the muscle regeneration regulator PAX7 and the synthetic rate‐limiting enzyme NMRK2 (Figure 5I). The expression of 10 key differential genes (Table S8) was determined by PCR, and the results showed that the expression of all target genes in the model group showed a significant down‐regulation trend compared with the normal control group (Tukey's HSD, *p* < 0.01) (Figure 5J). Among them, the expression levels of *MyHC*, *Pax7*, and *SIRT1* were the most significantly downregulated (Figure 5J). Following *L. johnsonii* Z‐RW intervention, the expression levels of the above genes were significantly restored (Tukey's HSD, *p* < 0.05), with the most significant up‐regulation of *MyHC*, *Pax7*, and *SIRT1*, In addition, Z‐RW treatment also markedly increased the transcription of other myogenesis‐ and muscle function–related genes, including *Nmrk*2, *Krt1*8, *ENT*1, and *Snd*1, generally shifting their expression toward the control level (Figure 5J). Consistent with these transcriptional changes, abundances of NAD⁺ and nicotinamide riboside (NR) were reduced in the model group, whereas Z‐RW intervention significantly elevated NAD⁺ and further increased NR relative to the model group, a result that was highly consistent with the preliminary proteomics analysis data (Figure 5J). Collectively, these results indicate that, under microbiota‐reconstituted conditions, *L. johnsonii* Z‐RW contributes to the remodeling of intestinal sugar–bile acid metabolic balance and is associated with the reactivation of Pax7/Nmrk2/SIRT1 signaling, thereby supporting a microbiota‐dependent gut–muscle axis underlying the muscle‐protective effects of chondroitin sulfate. Notably, mechanistic analyses were not performed in germ‐free mice receiving DCS alone, and therefore, mechanistic interpretations are restricted to microbiota‐reconstituted conditions.

## DISCUSSION

Glucocorticoid‐induced myopathy is a common metabolic muscle disorder caused by long‐term glucocorticoid use. It is characterized by muscle atrophy, loss of strength and endurance, and the symptoms are often difficult to reverse [11]. In the present study, we demonstrate that dietary DCS markedly alleviated glucocorticoid‐induced muscle atrophy in mice. Importantly, our data establish that these muscle‐protective effects are strictly dependent on the presence of gut microbiota, rather than resulting from direct action of DCS on skeletal muscle tissue. DCS administration failed to improve muscle phenotypes in germ‐free or microbiota‐depleted mice, whereas restoration of microbial communities, particularly through fecal microbiota transplantation or mono‐colonization with *Lactobacillus johnsonii* Z‐RW‐recapitulated muscle functional recovery. These findings position gut microbiota dependence as a prerequisite for DCS efficacy and define the gut–muscle axis as the dominant regulatory framework underlying the observed phenotype.

Previous studies have shown that polysaccharides not only act directly on the intestinal barrier but are also metabolized by the microflora into biologically active secondary products, which in turn affect distal tissue function. In this study, DCS was found to play an important role in regulating the “gut–muscle axis,” and different doses of DCS significantly promoted the colonization and proliferation of probiotic bacteria (e.g., *Lactobacillus johnsonii*) (Figure 2). Previous studies have shown that *L. johnsonii* has an important role in immunomodulation, gut health improvement, and prevention and treatment of metabolic diseases [12]. In the present study, we demonstrated that *L. johnsonii* Z‐RW significantly improved muscle functions, including muscle weight, running distance and grip strength, in the GIM (glucocorticoid‐induced myopathy) model of sterile mice by strain isolation and single‐strain gavage experiments. Colony‐derived secondary bile acids (e.g., 7‐KLCA and UDCA) promote muscle mitochondrial function and protein synthesis via TGR5, alleviating the inflammatory muscle atrophy phenotype. However, the specific mechanisms of interaction between gut flora and host muscle function in the context of GIM have not been systematically elucidated. In this study, intestinal metabolome analysis revealed that *L. johnsonii* Z‐WR, helped reverse the imbalance of metabolic homeostasis in skeletal muscle by remodeling the intestinal metabolic profile, up‐regulating the levels of free bile acids, and down‐regulating the metabolism of sugars. This was reflected by the restored mitochondrial function and the increased ATP synthesis, thus effectively alleviating the symptoms of myopathy. These results suggest that DCS and its mediated alteration of intestinal metabolites may target skeletal muscle metabolic dysfunction through a bidirectional “gut–muscle axis” regulatory mechanism, thereby ameliorating the symptoms of myopathy.

Notably, muscle phenotypic recovery was consistently associated with the accumulation of free and secondary bile acids following *L. johnsonii* Z‐RW colonization, whereas administration of DCS alone failed to elicit comparable effects in germ‐free mice. These findings indicate that the beneficial effects of DCS on muscle function are dependent on microbiota‐mediated metabolic transformation rather than direct action on host tissues. Although previous studies have suggested that bile acids may influence skeletal muscle metabolism through bile acid‐responsive signaling pathways, including FXR and TGR5 [13, 14], the present study does not directly interrogate receptor activation or downstream signaling events. Instead, the coordinated occurrence of bile acid remodeling and restoration of muscle metabolic and regenerative programs supports a microbiota‐dependent metabolic linkage between intestinal bile acid profiles and muscle function. In line with this interpretation, secondary bile acids have been reported to engage FXR‐ and TGR5‐associated signaling pathways in metabolic tissues, providing a biologically plausible context for the observed regulation of muscle‐associated proteins such as Nmrk2 and Pax7 in the present study. Nmrk2, a key enzyme in the NAD⁺ biosynthesis pathway, contributes to mitochondrial function and muscle repair [15]; while PAX7 is a well‐established marker of satellite cell activation and myogenic regeneration [14]; and KRT18 may support the integrity of muscle tissues by maintaining the structural stability of muscle fibers. These results further validate the critical role of DCS in alleviating glucocorticoid‐induced muscle atrophy. These results support secondary bile acids as candidate metabolic mediators linking *L. johnsonii* Z‐RW–associated metabolic remodeling to muscle phenotypic improvement through the gut–muscle axis, providing functional support for the involvement of metabolite remodeling in muscle regulation rather than definitive causal attribution to individual metabolites. In order to further examine whether changes in metabolite availability are functionally linked to muscle phenotypes, in vivo metabolite supplementation experiments were performed, in which representative sugars and bile acids were administered to mice. These experiments demonstrated that modulation of metabolite availability was sufficient to induce reproducible changes in muscle‐related phenotypes. However, such findings provide functional support for the involvement of metabolite remodeling in muscle regulation and do not establish definitive causality at the level of individual metabolites or their direct molecular targets. These metabolite supplementation experiments were not intended to establish strict causal or quantitative relationships, but rather to provide supportive evidence consistent with the microbiota‐dependent metabolic framework identified in this study.

In order to elucidate the mechanism of *L. johnsonii* Z‐RW in bile acid metabolism, whole genome sequencing technology was employed in this study, which identified the presence of the *bshA* gene, indicating that this strain harbors genetic potential for bile salt hydrolase‐associated activity, rather than direct genetic evidence of functional necessity. The gene *bshA* encodes bile salt hydrolase (BSH), an enzyme with a specific function of hydrolyzing bound bile salts, such as taurine‐bound bile salts or glycine‐bound bile salts [16]. In the presence of this enzyme, bound bile salts are broken down into free bile acids and the corresponding amino acids, such as taurine or glycine [17]. BSH precisely severs the amide bonds of bile salts to effectively unbind the bile acids from the amino acids, thereby promoting the presence of bile acids in their free form. Free bile acids, a crucial class of signaling molecules, exert fine regulation of physiological processes such as lipid metabolism, glucose metabolism, and energy homeostasis through activation of nuclear receptors (e.g., FXR, or farnesoid X receptor) or G‐protein‐coupled receptors (e.g., TGR5) in the host organism [18, 19]. Results of in vitro and in vivo culture experiments showed that *L. johnsonii* Z‐RW significantly reduced the levels of bound primary bile acids while elevating the levels of multiple secondary bile acids. Consistent with this genomic annotation, these bile acid deconjugation patterns support the involvement of BSH‐associated bile acid metabolism at the strain level. However, the present study does not include genetic manipulation of *bshA* (e.g., knockout or overexpression) to formally establish its necessity. Therefore, the contribution of BSH is interpreted within a functional and metabolic context rather than as a gene‐specific causal determinant. Deoxycholic acid has been shown to improve exercise endurance and muscle strength in mice [20]. The present study further revealed that several secondary bile acids, such as TDCA and HCA, β‐MCA, UDCA, and 7‐KLCA, may also be involved in the process of myopathy remission. In combination with the dysregulated bile acid profile common in patients with metabolic myopathy, these results support secondary bile acids as candidate metabolic mediators linking *L. johnsonii* Z‐RW colonization to muscle phenotypic improvement through the gut–muscle axis, providing a metabolic context for the observed effects of DCS.

In summary, this study delineates a microbiota‐dependent metabolic framework through which dietary chondroitin sulfate modulates skeletal muscle function under glucocorticoid‐induced myopathy. By integrating microbiome, metabolome, and proteome analyses with controlled microbial reconstitution models, we demonstrate that DCS activates the gut–muscle axis through coordinated remodeling of sugar utilization and bile acid metabolism, with *L. johnsonii* Z‐RW as a key contributing mediator. These findings provide mechanistic insight into how functional polysaccharides interface with gut microbial metabolism to influence distal tissue physiology, and they establish a conceptual foundation for future microbiota‐oriented strategies to mitigate muscle wasting. While this study focuses on stabilized endpoint phenotypes and does not resolve temporal dynamics or receptor‐level causality, future work combining strain‐specific genetic approaches, bile acid receptor‐oriented analyses, longitudinal microbiome and metabolite monitoring, and long‐term safety assessment will be essential to advance the mechanistic resolution and translational potential of DCS‐ or microbiota‐based strategies for muscle wasting disorders.

## METHODS

### Preparation and structural characterization of chondroitin sulfate (DCS)

Source of chondroitin sulfate: chondroitin sulfate used in this study was derived from duck bones, with a molecular weight of 8.23 kDa. It was successfully isolated and purified from duck bone by‐products through enzymatic treatment using chondroitinase. Structural characterization confirmed that low molecular weight chondroitin sulfate (DCS) consists of N‐acetylgalactosamine (GalNAc) and glucuronic acid (GlcUA) linked by β−1,3 and β−1,4 glycosidic bonds, forming a repeating disaccharide unit (→4)‐β‐d‐GlcpA‐(1 → 3)‐β‐d‐GalpNAc‐(1 → ). Disaccharide composition analysis identified GlcUA(2S)β1‐3GalNAc (10.00%), GlcUAβ1‐3GalNAc(4S) (30.21%), GlcUAβ1‐3GalNAc(6S) (14.86%), and GlcUA(2S)β1‐3GalNAc(4S) (44.93%) units.

### Animal experiments and model development

Fifty 7‐week‐old male SPF‐grade C57BL/6J mice (Beijing VTLH Laboratory Animal Technology Co., Ltd.) were selected and housed under conditions of 27 ± 1°C and a 12‐h light‐dark cycle. Mice were provided ad libitum access to standard chow and water. After a 1‐week acclimation period, the experiment commenced. Mice were randomly divided into five groups (10 mice per group): control group, model group, and low‐dose (100 mg/kg body weight), medium‐dose (200 mg/kg body weight), and high‐dose (400 mg/kg body weight) DCS intervention groups. During the pretreatment phase, control and model groups received daily oral administration of 0.1 mL saline solution, while the remaining groups received DCS solution at their respective doses via oral gavage for two consecutive weeks.

Following pretreatment, all mice except the control group were subjected to dexamethasone (DXMS)‐induced myasthenia gravis model: intraperitoneal injections of 25 mg/kg/day for the first 2 weeks, followed by 10 mg/kg/day for 5 weeks. At the conclusion of the experiment, all mice were euthanized, and blood samples were collected for hematological parameter analysis. The harvested tissues and organs were divided into two portions: one fixed in 10% neutral formalin for subsequent histological staining; the other rapidly frozen in liquid nitrogen for preservation, to be used for RNA and protein extraction and subsequent molecular biological analysis.

All experiments were conducted in accordance with the National Institutes of Health Guide for the Care and Use of Laboratory Animals and were approved by the Ethics Committee of the Department of Laboratory Animal Science (Approval No.: IACUC‐20231103‐02, IACUC‐20240922‐02, IACUC‐20250319‐02).

### Experimental indicator analysis and detection

This study employed multiple methods to evaluate muscle function and physiological status in mice: Grip strength was measured using a small animal dynamometer, following the protocol of Brooks et al. (1988) [21]. Following the protocol of Lerman et al. (2002) [22], exercise endurance was assessed using an electric treadmill. The Morris water maze test was conducted according to the procedure described by Vorhees et al. (2006) [23] to evaluate spatial learning and memory abilities. After the experiments, muscles were dissected and weighed for wet weight determination, employing the method referenced in Grounds et al. (2008) [24].

At the histological level, to assess muscle regenerative capacity, we performed H&E staining on gastrocnemius muscle sections and calculated the myofibrillar fusion index following method described by Briguet et al. (2004) [25]. Additionally, in vivo imaging experiments were conducted using the protocol of Hüttemeister et al. (2024) [26]. Tissue immunofluorescence detection followed the protocol of Esper et al. (2023) [27]. Fluorescent signal quantification was performed using the Aipathwell® system by setting HSI thresholds, with at least five fields of view at 200 × magnification analyzed per group.

### In vitro utilization of fecal microbiota for DCS

Collect fresh feces from mice in different treatment groups. Prepare the microbial inoculum by adding 1.0 g of feces to 10 mL of sterile PBS (pH 7.2–7.4). Centrifuge at 600 × *g* at 4°C for 5 min to remove solids, then use the supernatant as the microbial inoculum. The inoculum was added at a 3% volume to GMM medium supplemented with DCS as the carbon source and fermented anaerobically at 37°C. Samples were collected at different time points, centrifuged at 8000 × *g* for 15 min at 4°C to collect the supernatant, and DCS utilization relative to 0 h was calculated [28].

### Co‐culture of DCS and bacterial strains

After thawing cryopreserved strains, passage them through solid and liquid media until the logarithmic phase. Wash with PBS and adjust the bacterial suspension concentration (OD600 = 0.6–0.7). Inoculate the strains at a 1:10 ratio into DM medium using DCS as the carbon source, and incubate under anaerobic conditions at 37°C for 7 days. The fermentation broth was collected by centrifugation at 8000 × *g* and 4°C for 10 min to obtain the supernatant [29]. The effect of DCS on bacterial growth and proliferation was evaluated using the viable cell count method.

### Strain adhesion rate assay

Seed Caco‐2 (cells into a 24‐well plate (approximately 2.5 × 10*5 cells/well). Once a confluent monolayer is established, add 100 μL of bacterial suspension to each well and incubate at 37°C for 1.5 h. Discard the medium, wash five times with PBS, digest with 0.1% trypsin‐0.02% EDTA for 10 min, resuspend in PBS, and perform a serial dilution. Plate the dilution onto MRS agar plates and incubate at 37°C. Count the colonies. Adhesion rate (%) = Adhered bacteria/Inoculated bacteria × 100%. Each group included three replicates, with three independent replicates performed [30].

### Western blot

Proteins were extracted from lysed tissues, separated by SDS‐PAGE, and transferred to membranes. Primary and HRP‐labeled secondary antibodies were incubated, with β‐actin as the internal control. Signals were analyzed using ImageJ.

### RT‐PCR

Total RNA was extracted using the TRIzol method. After quality control, it was reverse transcribed into cDNA. The SYBR Green method was employed for detection, with β‐actin as the internal control, Gene primer sequences are shown in Table S8. Results were calculated using the 2^^−ΔΔCt^ method.

### Gut microbiome analysis

Total DNA was extracted from gut content samples stored at −80°C using a commercial kit. Following quality control, 16S rRNA V3–V4 region was amplified. Library preparation and Illumina NovaSeq. 6000 platform sequencing (2 × 250 bp) was outsourced to Nanjing Paisennuo Gene Technology Co., Ltd. Sequencing data underwent standard processing following Bolyen's method [31]. Sequence data from all 16S rRNA sequencing experiments have been deposited in the National Center for Biotechnology Information Sequence Read Archive under accession number PRJNA1327095.

### Gut metabolomics analysis

After methanol extraction and pretreatment, intestinal samples were submitted to Nanjing Paisennuo Gene Technology Co., Ltd. for non‐targeted metabolomics analysis. Chromatographic separation was performed using UPLC, with mass spectrometry detection conducted on an Orbitrap high‐resolution platform in simultaneous positive and negative ion modes. Raw data were processed using MS‐DIAL, and metabolite annotation was based on the HMDB and LIPID MAPS databases.

### Proteomics analysis

Mouse tissue lysates were quantified and enzymatically digested, then submitted to Hebei Xiankang Biotechnology Co., Ltd. (Hebei, China) for DIA‐based proteomics using an Orbitrap Astral platform. Data were searched against the UniProt Mus musculus database and analyzed with DIA‐NN software [32] with FDR ≤ 1% control, following established DIA workflows [33].

### Isolation of strains and genome sequencing

Following established methodologies [34, 35], target strains were isolated from mouse feces. Their genomic information was obtained and submitted to NCBI (PRJNA1280473). The strains are deposited at CGMCC (No. 32588).

### Strain and animal fecal microbiota transplantation experiments

To clarify the independent role of *Lactobacillus johnsonii* in alleviating myasthenia gravis, a mouse model was established [36], with a single‐strain group and an FMT group established. Single‐strain group received oral gavage of bacterial suspension (1 × 10⁹ CFU/mL, 0.2 mL/day) for 14 days; FMT group used donors from mice subjected to DCS intervention. Prior to transplantation, mice received daily oral administration of broad‐spectrum antibiotics (ampicillin, neomycin, vancomycin, metronidazole) for 3 consecutive days to eradicate endogenous microbiota [37]. Following a 12‐h fasting period, daily oral administration of 0.2 mL fecal microbiota suspension commenced. Post‐intervention measurements included muscle mass, running distance, grip strength, swimming speed, and histopathology. Intestinal samples were collected for proteomic and metabolomic analysis.

### In vitro functional assay

*Lactobacillus johnsonii* Z‐RW (CGMCC No. 32588, stored at –80°C in glycerol) was anaerobically passaged three times in MRS medium (37°C, 24 h) to ensure stable activity. Subsequently, a 2% inoculum (OD600 = 0.6 ± 0.05) was added to selective media containing different carbon sources or bile acid substrates [xylobiose, mannose, erythrose, xylose, taurodeoxycholic acid (TDCA), taurocholic acid (TCA), glycochenodeoxycholic acid (GCDCA), hyocholic acid (HCA), and mixed bile acids (1:1:1:1, w/w)] and cultured under strict anaerobic conditions at 37°C for 24 h. Viable cell counts (CFU/mL) were determined by the dilution plate method, and substrate consumption and conversion were assessed.

### Targeted metabolomics analysis

To investigate key metabolic shifts before and after bacterial metabolism, targeted metabolomics detection was performed. Bile acid quantification primarily followed the method described [38]. After sample processing, separation was achieved via gradient elution on a BEH C18 column (2.1 × 100 mm, 1.7 μm; Waters, USA) using an ultra‐performance liquid chromatography‐tandem mass spectrometry (UPLC‐MS/MS) system (Waters Xevo TQ‐S). Mass spectrometry employed Multiple Reaction Monitoring (MRM) mode combined with internal standardization for absolute quantification of bile acids. Additionally, SCFAs were detected following the protocol [39]. Samples were derivatized with 3‐nitrophenylhydrazine (3‐NPH) and analyzed on an SCIEX Triple Quad™ 6500 + LC‐MS/MS system. Quantification of SCFAs such as acetic acid, propionic acid, and butyric acid was performed using MRM mode with external standard calibration curves, while data quality was monitored using quality control (QC) samples.

### Metabolite supplementation experiment

To elucidate the relative contributions of direct metabolite action and *L. johnsonii* Z‐RW‐mediated metabolite conversion in alleviating muscle weakness, a total of 24 groups were established using germ‐free mice. These included a control group (unmodeled mice), a sarcopenia model group, single‐sugar groups, a mixed‐sugar intervention group (xylose: mannose: erythrose: xylobiose = 1:1:1:1, w/w), single bile acid groups, a mixed bile acid intervention group (TDCA: GCDCA: TCA: HCA = 1:1:1:1, w/w), and corresponding intervention groups supplemented with *L. johnsonii* Z‐RW. The muscle weakness modeling method was as previously described, with six mice per group. Post‐intervention physiological parameters, including muscle weight, running distance, grip strength, and swimming speed, were assessed using the same methods as described previously.

### Statistical analysis

All statistical analyses were performed using the R 4.4 software environment [40], unless specified otherwise. Differential analysis was conducted using the R package “EasyStat” (v 0.1.0) [41]. Data normality was assessed using the Shapiro‐Wilk test. For normally distributed data, differences between two groups were evaluated using independent samples *t*‐tests, while differences among three or more groups were analyzed using one‐way ANOVA followed by Tukey's Honestly Significant Difference test for post hoc pairwise comparisons. For non‐normally distributed data, differences between two groups were assessed using the Wilcoxon rank‐sum test, while differences among three or more groups were evaluated using the Kruskal–Wallis test followed by Dunn's test with Benjamini–Hochberg false discovery rate (FDR) correction for post hoc pairwise comparisons. Statistical significance was defined as *p* < 0.05 for all tests. Linear regression analysis was performed using the “lm” function from the “stats” package (v 4.3.0) [40], with R‐squared (*R*²) and *p*‐values reported. Correlation plots were generated using the “ggpubr” package (v 0.6.0) [42]. For multi‐omics data analysis, principal coordinates analysis was first applied to extract key features. Subsequently, partial least squares path modeling was employed to construct a comprehensive model exploring the contributions of different omics datasets to the improvement of muscle weakness phenotypes in mice.

### Microbiome analysis

Prior to alpha diversity analysis, sequence reads were rarefied to an even sampling depth across all samples using “vegan” package [43]. Beta diversity was assessed based on Bray–Curtis dissimilarity matrices calculated from normalized ASV relative abundances using same package. Resulting dissimilarity matrices were visualized through NMDS with 1000 random starts to ensure stability of the ordination. “randomForest” package (v 4.7.1) [44] was used to develop random forest models. Model performance was evaluated using out‐of‐bag error estimation. To evaluate dose‐dependent effects of drug treatments on microbial composition, we performed effect size analysis using “metafor” package (v 4.6.0). Differentially abundant taxa between treatment groups were identified using “edgeR” package (v 3.42.2) [45]. Significant enrichment or depletion was assigned to species with adjusted *p* < 0.05 and |log2 fold change| > 1 in comparisons with both control and model groups.

### Metabolite analysis

Raw metabolite peak areas were preprocessed in MetaboAnalyst (v 4.0.0) [46] through the following pipeline: (1) missing values were imputed using k‐nearest neighbors for compounds with <50% missingness, (2) data were normalized by sample median followed by Pareto scaling. Processed metabolomic matrix was subsequently analyzed in R. Metabolite clusters were visualized via hierarchical clustering using ComplexHeatmap (v 2.2.0) [47] with Euclidean distance and complete linkage methods. PLS‐DA was performed on metabolite data using “mixOmics” package (v 6.2.0). Significant separation between groups was confirmed by permutational multivariate analysis of variance (PERMANOVA) using the “vegan” package with Bray–Curtis distances and 999 permutations. To assess global covariation between microbial communities and metabolomic profiles, Procrustes analysis was performed on Bray–Curtis (microbiota) and Euclidean (metabolites) distance matrices using protest function in *vegan* package (v 2.6‐4), with significance tested via 999 permutations. For targeted interaction analysis, core microbiota (*Lactobacillus johnsonii*) were correlated with two biologically relevant metabolite classes (carbohydrates and bile acids) using *ggClusterNet* package (v 0.99.1) [48].

### Proteomic profiling and functional analysis

Protein enrichment analysis was performed using ClusterGVis for trend analysis and functional annotation. Specifically, clustering analysis was performed on the global protein expression profiles using K‐means and Mfuzz algorithms, which classified the data into six distinct expression modules. Gene Ontology (GO) enrichment analysis was conducted for the terms in each module using Fisher's exact test (FDR < 0.05), and KEGG pathway analysis was performed using the clusterProfiler package [49]. Module C6 was selected for further analysis based on its significant enrichment in pathways related to muscle system process. Within Module C6, differential expression analysis was performed to identify proteins that were significantly upregulated in the three treatment groups compared to the Model group, with a threshold of Log_2_ FC > 2 and *p*_adj_ < 0.05. This step yielded 35 differentially expressed proteins. To strictly identify proteins associated with phenotypic recovery, we applied a secondary filter to select proteins that maintained significantly higher expression levels in the Normal group compared to the Model group, narrowing the candidates to 23 proteins. Based on functional annotation and literature review focusing on muscle function, four key proteins were identified as final targets for validation.

## AUTHOR CONTRIBUTIONS

**Ruiyun Wu**: Methodology; validation; formal analysis; investigation; writing—review and editing; writing—original draft. **Tao Wen**: Formal analysis; investigation; writing—review and editing; writing—original draft. **Nan Shang**: Writing—review and editing; investigation; formal analysis; funding acquisition. **Penghao Xie**: Writing—review and editing; investigation; formal analysis. **Zhenyu Wang**: Investigation; funding acquisition. **Hang Li**: Investigation. **Shaobo Li**: Investigation. **Dequan Zhang**: Conceptualization; methodology; writing—review and editing; funding acquisition; supervision. All authors have read the final manuscript and approved it for publication.

## CONFLICT OF INTEREST STATEMENT

The authors declare no conflicts of interest.

## ETHICS STATEMENT

All the animal procedures were conducted in accordance with the National Institutes of Health Guide for the Care and Use of Laboratory Animals and were approved by the Ethics Committee of the Department of Laboratory Animal Science (Approval No.: IACUC‐20231103‐02, IACUC‐20240922‐02, IACUC‐20250319‐02).

## Supporting information


**Figure S1:** Microbiota‐independent evaluation of DCS safety and cellular effects.
**Figure S2:** Principal coordinate analysis (PCoA) based on Bray‐Curtis dissimilarity of protein profiles.
**Figure S3:** Line plot evaluating the number of protein expression clusters.
**Figure S4:** Expression of muscle regeneration–related proteins in gastrocnemius tissue after DCS intervention.
**Figure S5:** *Alpha* diversity of gut microbial communities across different treatments. Different lowercase letters indicated significant differences between groups based on Wilcoxon rank‐sum test (*p* < 0.05).
**Figure S6:** Non‐metric multidimensional scaling (NMDS) based on Bray–Curtis dissimilarity.
**Figure S7:** Determination of the optimal number of features via cross‐validation in the random forest model.
**Figure S8:** Correlation analysis between the relative abundance of *L. johnsonii* Z‐RW and the levels of sugars and bile acids in the murine gut.

**Table S1:** Effects of different concentrations of DCS on various physiological indicators in mice.
**Table S2:** DCS concentrations in mouse blood and tissues after oral gavage (0, 12, and 24 h).
**Table S3:** List of differentially expressed proteins (DEPs) identified in Module C6. DCS refers to the collective three treatment groups (Low, Medium, and High dosages).
**Table S4:** General toxicological parameters in female and male mice following acute oral administration of DCS.
**Table S5:** COG functional classification groups.
**Table S6:** Effects of metabolite supplementation on various physiological indicators in mice.
**Table S7:** Effects of strain supplementation on various physiological indicators in mice.
**Table S8:** Sequences of primers used in real‐time qPCR.

## Data Availability

The data that support the findings of this study are openly available in GitHub at https://github.com/xph007/Chondroitin. Sequence data from all 16S rRNA sequencing experiments have been deposited in the National Center for Biotechnology Information Sequence Read Archive (SRA) under accession number PRJNA1327095 (https://www.ncbi.nlm.nih.gov/bioproject/PRJNA1327095/). Complete genome sequence of *Lactobacillus johnsonii* Z‐RW was deposited at GenBank under accession nos. SUB 15504534 (https://www.ncbi.nlm.nih.gov/bioproject/PRJNA1280473). This strain has been deposited in the China Center of Industrial Culture Collection under accession number CGMCC No. 32588. The data and scripts used are saved in GitHub (https://github.com/xph007/Chondroitin). Supplementary materials (figures, tables, graphical abstract, slides, videos, Chinese translated version, and update materials) can be found in the online DOI or iMeta Science http://www.imeta.science/.
