# Supplementary material for: Chondroitin sulfate restores muscle mass via gut–muscle axis remodeling through sugar–bile acid metabolism reprogramming
Source: Imeta. 2026 Mar 12;5(2):e70118. doi: 10.1002/imt2.70118 (PMC13147937; doi:10.1002/imt2.70118)
Supplement: Supplementary file 1 — Figure S1: Microbiota‐independent evaluation of DCS safety and cellular effects. Figure S2: Principal coordinate analysis (PCoA) based on Bray‐Curtis dissimilarity of protein profiles. Figure S3: Line plot evaluating the number of protein expression clusters. Figure S4: Expression of muscle regeneration–related proteins in gastrocnemius tissue after DCS intervention. Figure S5: Alpha diversity of gut microbial communities across different treatments. Different lowercase letters indicated significant differences between groups based on Wilcoxon rank‐sum test (p < 0.05). Figure S6: Non‐metric multidimensional scaling (NMDS) based on Bray–Curtis dissimilarity. Figure S7: Determination of the optimal number of features via cross‐validation in the random forest model. Figure S8: Correlation analysis between the relative abundance of L. johnsonii Z‐RW and the levels of sugars and bile acids in the murine gut. [file IMT2-5-e70118-s002.docx]

**Supporting information to**

# **Chondroitin sulfate restores muscle mass via gut–muscle axis remodeling through sugar–bile acid metabolism reprogramming**

**Running title:** Microbiota-dependent muscle restoration by chondroitin sulfate

Ruiyun Wu^1#^, Tao Wen^2#^, Nan Shang^3#^, Penghao Xie^2^, Zhenyu Wang^1^, Hang Li^4^, Shaobo Li^1^, Dequan Zhang ^1*^

^1^Institute of Food Science and Technology, Chinese Academy of Agricultural Sciences, Integrated Laboratory of Processing Technology for Chinese Meat and Dish Products, Ministry of Agriculture and Rural Affairs, Beijing 100193, China.

^2^Jiangsu Provincial Key Lab for Organic Solid Waste Utilization, Jiangsu Collaborative Innovation Center for Solid Organic Wastes, Educational Ministry Engineering Center of Resource-saving fertilizers, Nanjing Agricultural University, Nanjing 210095, China.

^3^Collage of Engineering, China Agricultural University, Beijing 100083, China.

^4^Key Laboratory of Biomaterials of Guangdong Higher Education Institutes, Department of Biomedical Engineering, Jinan University, Guangzhou 510632, China.

**^#^**These authors contributed equally: Ruiyun Wu, Tao Wen, Nan Shang

*Corresponding Author: dequan_zhang0118@126.com (Dequan Zhang)

**
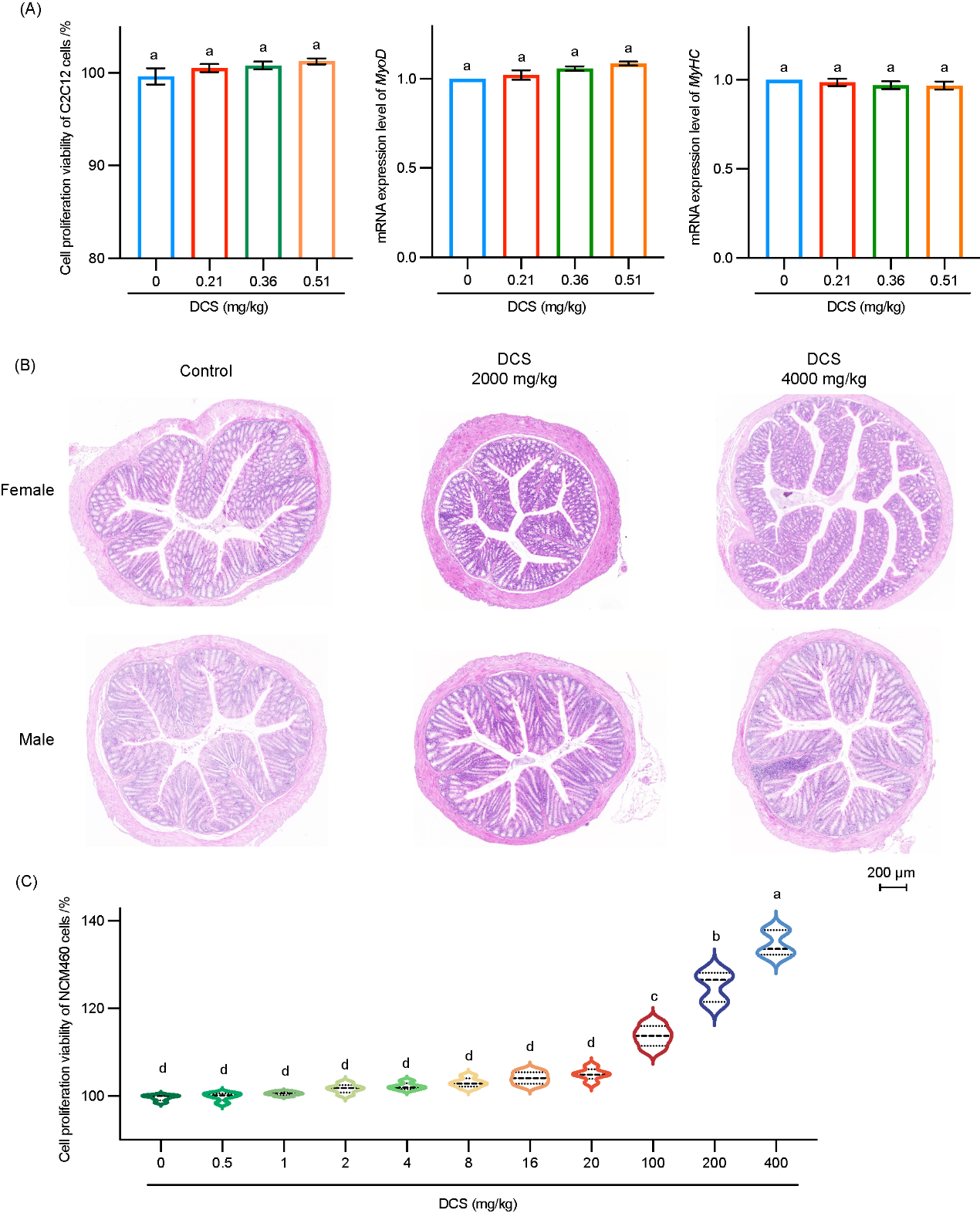
**

**Figure S1 Microbiota-independent evaluation of DCS safety and cellular effects.** (A) Effects of physiologically relevant DCS concentrations on C2C12 cells. (B) H&E staining of colon sections after acute oral DCS administration. (C) Effects of DCS on the viability of NCM460 cells. Different letters indicate significant differences (*p* < 0.05).


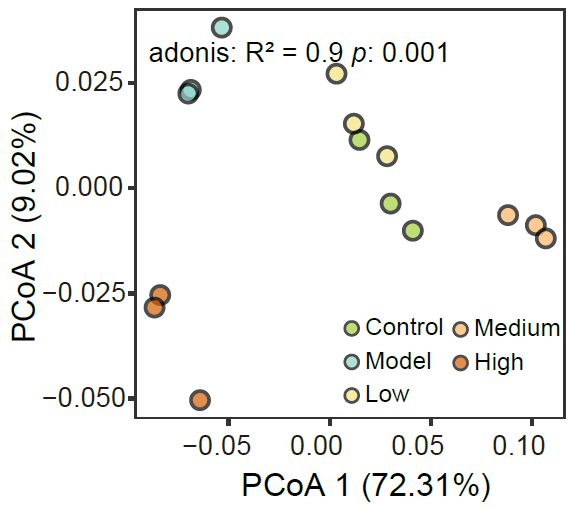


**Figure S2 Principal coordinate analysis (PCoA) based on Bray-Curtis dissimilarity of protein profiles.**


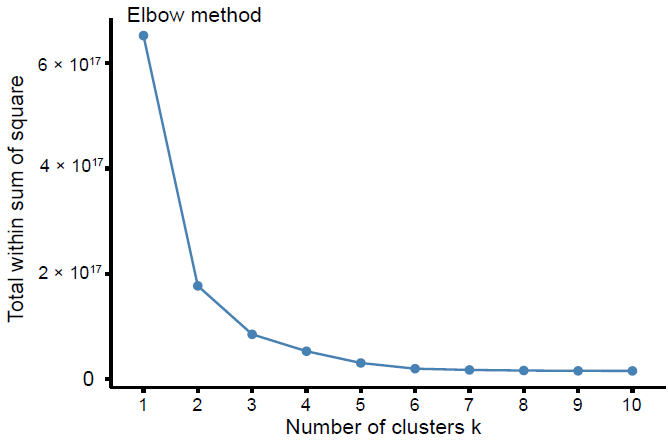


**Figure S3 Line plot evaluating the number of protein expression clusters.**


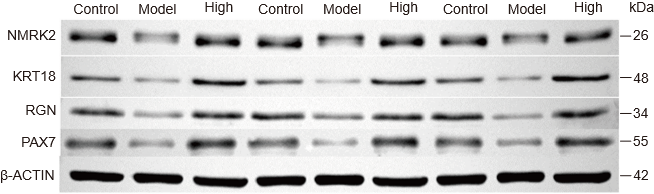


**Figure S4 Expression of muscle regeneration–related proteins in gastrocnemius tissue after DCS intervention.**


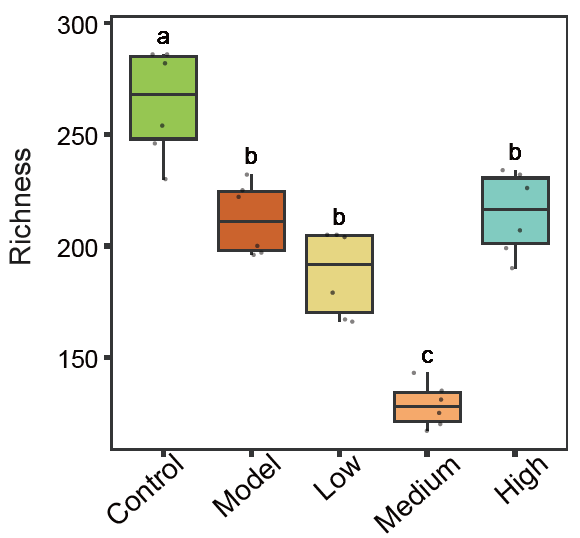


**Figure S5 *Alpha* diversity of gut microbial communities across different treatments.** Different lowercase letters indicated significant differences between groups based on Wilcoxon rank-sum test (*p* < 0.05).


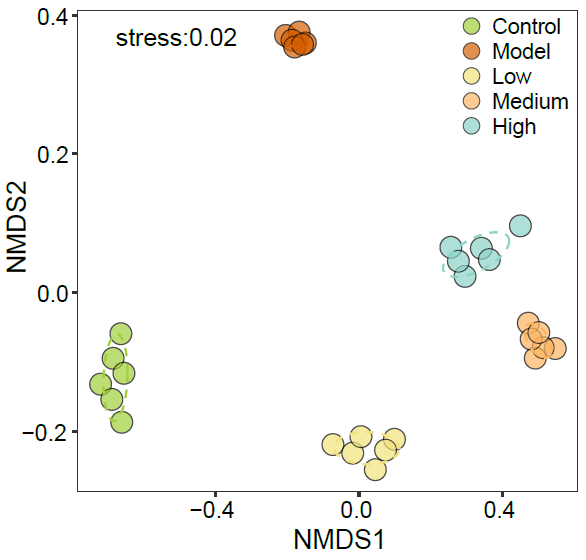


**Figure S6 Non-metric multidimensional scaling (NMDS) based on Bray–Curtis dissimilarity.**


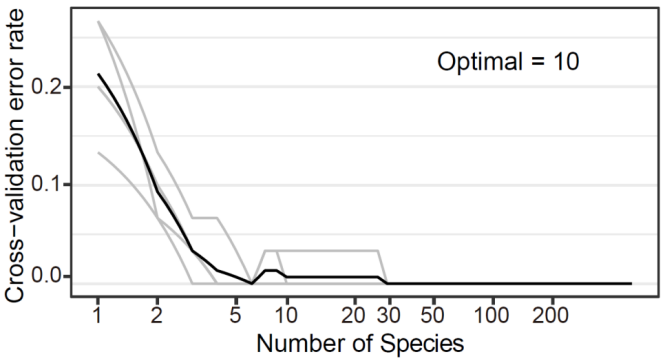


**Figure S7 Determination of the optimal number of features via cross-validation in the random forest model.**


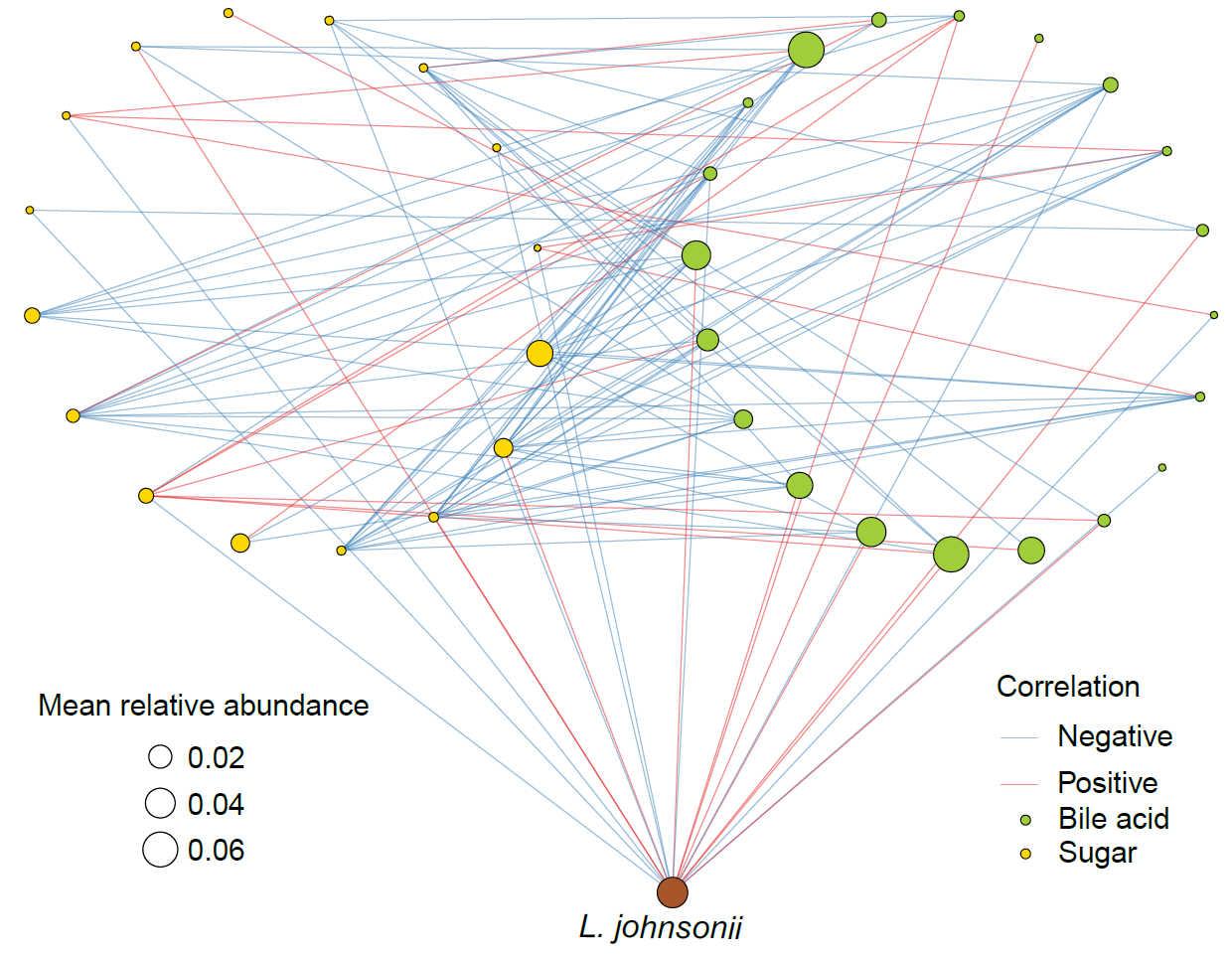


**Figure S8 Correlation analysis between the relative abundance of *L. johnsonii* Z-RW and the levels of sugars and bile acids in the murine gut.**
